# Supplementary material for: Dermal Absorption of Sesquiterpene Lactones from Arnica Tincture
Source: Pharmaceutics. 2022 Mar 29;14(4):742. doi: 10.3390/pharmaceutics14040742 (PMC9027632; doi:10.3390/pharmaceutics14040742)
Supplement: Supplementary file 1 [file pharmaceutics-14-00742-s001.zip › pharmaceutics-1630976_proofreading_SM checked.pdf]

pharmaceutics

MDPI

Supplementary Materials

Dermal Absorption of Sesquiterpene Lactones from Arnica Tincture

Franziska M. Jürgens, Fabian C. Herrmann, Sara M. Robledo and Thomas J. Schmidt

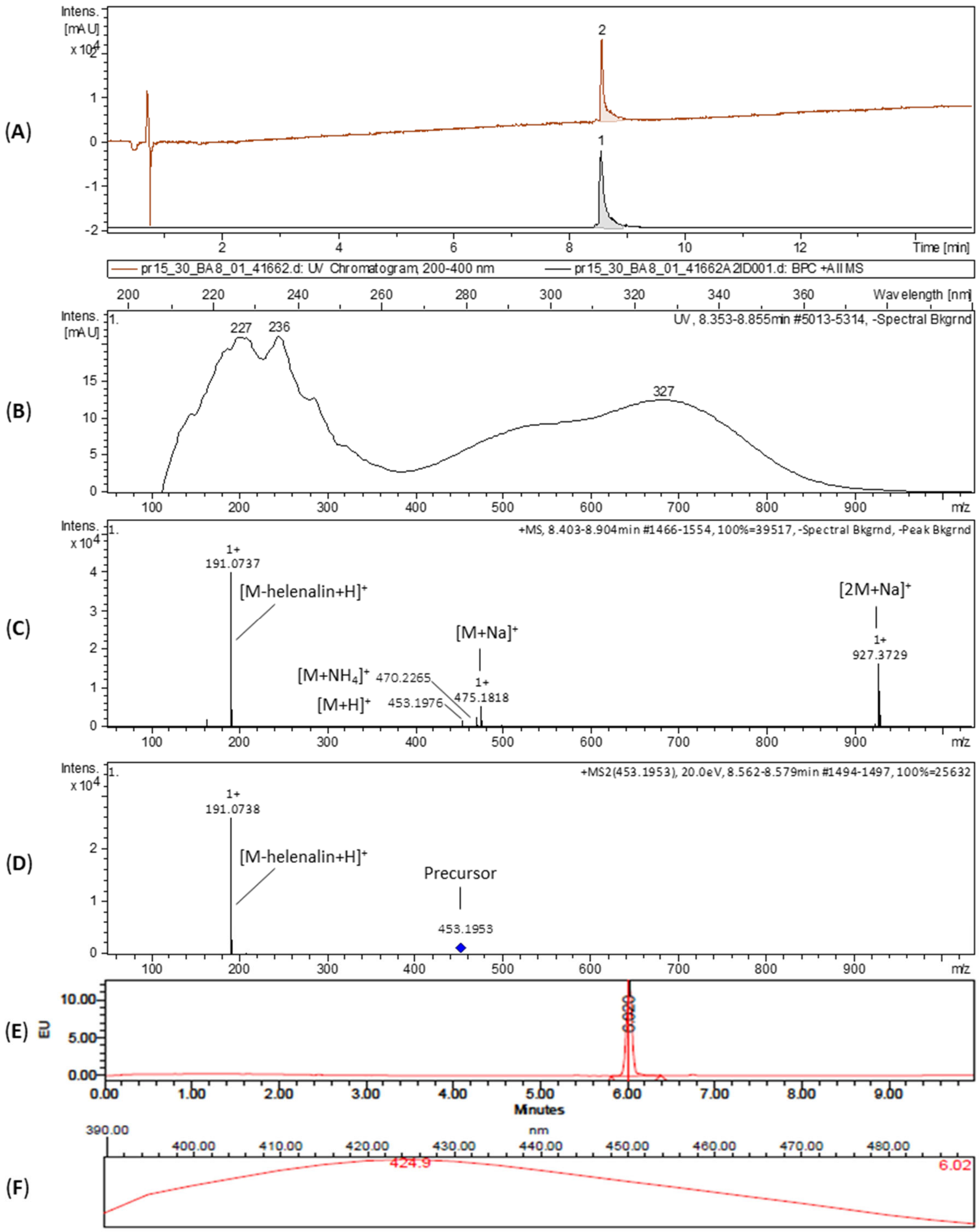

Figure S1. (**A**–**D**) UHPLC-DAD-HRMS analysis of H-DMCA. (**A**) Base Peak Chromatogram (black, *m*/*z* 50–1500) and UV chromatogram (red, 200–400 nm), (**B**) UV absorption spectra, (**C**) full MS spectra with signals of the protonated molecule $[M+H]^+$, the ammonium adduct $[M+NH_4]^+$, two sodium adducts $[M+Na]^+$ and $[2M+Na]^+$ and a fragment resulting from ester cleavage $[M\text{-helenalin}+H]^+$ and (**D**) MS/MS spectra with $[M+H]^+$ as precursor ion. (**E**–**F**) UHPLC-FLD analysis of H-DMCA. (**E**) PDA Max Plot chromatogram (maximum spectral absorbance measured at each time point) with excitation at 327 nm and emission at 390–490 nm. (**F**) Emission spectra (390–490 nm) of H-DMCA (Rt = 6.020 min).

Table S1. ^{1}H- and ^{13}C-NMR (600/ 150 MHz, $CDCl_3$) data of H-DMCA: chemical shifts (δ), types of carbon (Cx), multiplet analysis (mult.) and coupling constants (*J*).

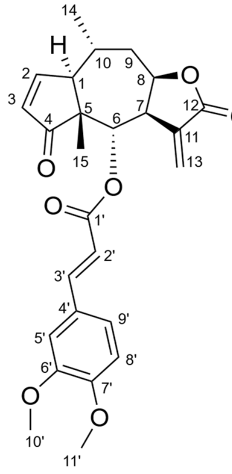

Pos.	δC [ppm], Cx[1]	δH [ppm], mult.[2], *J* [Hz]
1	53.28, CH	3.13, td, 11.3, 1.9
2	162.13, CH	7.69, dd, 6.1, 2.0
3	130.03, CH	6.10, dd, 6.1, 3.0
4	204.09, Cq	-
5	55.51, Cq	-
6	77.74, CH	5.51, d, 1.5
7	47.75, CH	3.62, ddt, 7.5, 2.7, 1.4
8	78.13, CH	4.90, td, 7.4, 2.4
9	40.16, CH2	2.42, ddd, 15.3, 7.1, 3.4
		1.80, ddd, 15.4, 8.7, 2.4
10	26.07, CH	2.20, m
11	137.48, Cq	-
12	169.49, Cq	-
13	125.16, CH2	6.48, d, 2.8
		6.22, d, 2.5
14	20.15, CH3	1.30, d, 6.7
15	18.44, CH3	1.04, s
1′	166.25, Cq	-
2′	114.73, CH	6.16, d, 15.7
3′	146.05, CH	7.58, d, 15.8
4′	126.58, Cq	-
5′	109.59, CH	7.00, d, 2.0
6′	149.17, Cq	-
7′	151.40, Cq	-
8′	111.00, CH	6.85, d, 8.4
9′	122.71, CH	7.07, dd, 8.4, 2.1
10′	55.88, CH3	3.91, s
11′	55.93, CH3	3.92, s

[1] Cx: CH, CH_2, CH_3 or Cq (quaternary carbon)

[2] mult.: singlet (s), doublet (d), triplet (t), multiplet (m)

Table S2.: Quantified STL amounts [µg] and percentage of applied amount [%] in skin wash solutions (W), skin extracts (S) and receptor fluids (R) of pig skin (P), human skin A (HA) and human skin B (HB) samples (mean ± standard deviation, *n*=6).

[µg]	WP	WHA	WHB	SP	SHA	SHB	RP	RHA	RHB
DH	0.94 ± 0.29	0.85 ± 0.37	0.84 ± 0.2	1.88 ± 1.23	2.03 ± 1.23	3.45 ± 1.41	3.66 ± 3.35	1.81 ± 1.64	4.21 ± 2.44
H	0.27 ± 0.09	0.25 ± 0.07	0.09 ± 0.05	0.48 ± 0.08	0.62 ± 0.13	0.56 ± 0.06	0.31 ± 0.23	1.61 ± 1.35	3.17 ± 1.06
DHac	0.16 ± 0.04	0.14 ± 0.04	0.04 ± 0.02	0.40 ± 0.12	0.41 ± 0.14	0.33 ± 0.11	0.93 ± 0.55	1.30 ± 0.41	4.80 ± 0.60
Hac	0.37 ± 0.12	0.32 ± 0.10	0.08 ± 0.04	0.50 ± 0.19	0.57 ± 0.15	0.48 ± 0.12	1.41 ± 0.57	2.24 ± 0.55	6.50 ± 1.06
DHma	0.29 ± 0.09	0.26 ± 0.09	0.04 ± 0.03	1.01 ± 0.13	1.12 ± 0.22	0.71 ± 0.28	1.22 ± 0.72	2.43 ± 0.56	6.92 ± 1.58
DHib	0.68 ± 0.21	0.62 ± 0.20	0.10 ± 0.06	2.20 ± 0.30	2.38 ± 0.53	1.58 ± 0.34	3.18 ± 1.76	5.91 ± 1.44	14.67 ± 1.49
Hma	0.70 ± 0.21	0.66 ± 0.21	0.11 ± 0.06	1.82 ± 0.53	2.17 ± 0.44	1.40 ± 0.24	2.38 ± 0.83	4.70 ± 1.02	10.98 ± 0.84
Hib	0.63 ± 0.20	0.57 ± 0.20	0.09 ± 0.05	1.48 ± 0.40	1.71 ± 0.36	1.07 ± 0.19	2.00 ± 0.66	3.96 ± 0.84	9.14 ± 0.63
DHt	0.19 ± 0.06	0.17 ± 0.05	0.03 ± 0.02	0.78 ± 0.12	0.87 ± 0.14	0.58 ± 0.15	0.65 ± 0.35	1.43 ± 0.34	3.96 ± 0.68
Ht	0.74 ± 0.22	0.70 ± 0.22	0.14 ± 0.07	2.63 ± 0.74	3.18 ± 0.48	2.25 ± 0.26	2.10 ± 0.71	4.42 ± 0.93	10.58 ± 1.15
DHmb	0.33 ± 0.10	0.29 ± 0.10	0.05 ± 0.03	1.45 ± 0.20	1.50 ± 0.22	0.98 ± 0.11	1.29 ± 0.66	2.67 ± 0.59	6.33 ± 0.67
Hmb	0.97 ± 0.29	0.92 ± 0.31	0.16 ± 0.09	3.56 ± 0.90	3.87 ± 0.58	2.43 ± 0.38	2.84 ± 1.00	5.70 ± 1.67	13.91 ± 2.88

DHs	2.58 ± 0.78	2.34 ± 0.84	1.11 ± 0.34	7.71 ± 1.55	8.31 ± 2.26	7.63 ± 2.29	10.92 ± 6.87	15.54 ± 5.57	40.89 ± 5.49
Hs	3.68 ± 1.10	3.42 ± 1.11	0.66 ± 0.35	10.47 ± 2.71	12.13 ± 2.06	8.19 ± 1.08	11.03 ± 3.90	22.63 ± 5.76	54.28 ± 5.90

**[%]**	**WP**	**WHA**	**WHB**	**SP**	**SHA**	**SHB**	**RP**	**RHA**	**RHB**
DH	2.9 ± 0.9	2.6 ± 1.1	2.6 ± 0,6	5.7 ± 3.7	6.2 ± 3.8	10.5 ± 4.3	11.2 ± 10.2	5.5 ± 5.0	12.8 ± 7.4
H	3.5 ± 1.1	3.3 ± 0.9	1.1 ± 0.6	6.3 ± 1.1	8.2 ± 1.7	7.4 ± 0.8	4.1 ± 3.1	21.1 ± 17.7	41.6 ± 13.8
DHac	2.8 ± 0.7	2.4 ± 0.8	0.7 ± 0.3	7.1 ± 2.2	7.2 ± 2.4	5.9 ± 2.0	16.5 ± 9.8	23.1 ± 7.3	85.5 ± 10.7
Hac	3.0 ± 1.0	2.6 ± 0.8	0.6 ± 0.3	4.0 ± 1.5	4.6 ± 1.2	3.8 ± 1.0	11.3 ± 4.6	18.0 ± 4.4	52.4 ± 8.5
DHma	2.9 ± 0.9	2.6 ± 0.8	0.4 ± 0.3	10.0 ± 1.3	11.1 ± 2.2	7.0 ± 2.7	12.0 ± 7.1	24.1 ± 5.6	68.6 ± 15.6
DHib	2.9 ± 0.9	2.7 ± 0.9	0.4 ± 0.3	9.4 ± 1.3	10.2 ± 2.3	6.8 ± 1.4	13.6 ± 7.5	25.3 ± 6.2	62.9 ± 6.4
Hma	2.0 ± 0.6	1.9 ± 0.6	0.3 ± 0.2	5.3 ± 1.5	6.3 ± 1.3	4.0 ± 0.7	6.9 ± 2.4	13.6 ± 2.9	31.7 ± 2.4
Hib	2.5 ± 0.8	2.2 ± 0.8	0.3 ± 0.2	5.8 ± 1.6	6.6 ± 1.4	4.2 ± 0.8	7.8 ± 2.5	15.4 ± 3.3	35.5 ± 2.4
DHt	3.1 ± 1.0	2.8 ± 0.9	0.5 ± 0.3	12.6 ± 1.9	14.2 ± 2.3	9.4 ± 2.4	10.6 ± 5.7	23.2 ± 5.5	64.3 ± 11.1
Ht	1.9 ± 0.6	1.8 ± 0.6	0.4 ± 0.2	6.9 ± 1.9	8.3 ± 1.3	5.9 ± 0.7	5.5 ± 1.9	11.5 ± 2.4	27.6 ± 3.0
DHmb	2.9 ± 0.9	2.6 ± 0.9	0.5 ± 0.3	12.9 ± 1.8	13.3 ± 2.0	8.7 ± 0.9	11.4 ± 5.8	23.6 ± 5.2	56.0 ± 6.0
Hmb	1.8 ± 0.5	1.7 ± 0.6	0.3 ± 0.2	6.6 ± 1.7	7.2 ± 1.1	4.5 ± 0.7	5.3 ± 1.9	10.6 ± 3.1	25.7 ± 5.3

DHs	2.9 ± 0.9	2.6 ± 0.9	1.2 ± 0.4	8.6 ± 1.7	9.3 ± 2.5	8.5 ± 2.6	12.2 ± 7.7	17.4 ± 5.0	45.8 ± 6.2
Hs	2.1 ± 0.6	2.0 ± 0.6	0.4 ± 0.2	6.1 ± 1.6	7.0 ± 1.2	4.7 ± 0.6	6.4 ± 2.3	13.1 ± 3.3	31.4 ± 3.4

1. Method Validation UHPLC-HRMS System

1.1 Full validation

The UHPLC-HRMS method was validated by applying ICH Guideline M10. Full method validation was carried out with an injection volume of 2 µL, later injection volume was increased to 200 µL and consequently partial validation was performed as demanded by ICH guideline M10. With an injection volume of 2 µL, the limit of detection (LOD, S/N > 3) was determined to be 0.1 µg/mL and the lowest limit of quantification (LLOQ, S/N ≥ 10) was 1.0 µg/mL for all analytes. The calibration range was defined by LLOQ and the upper limit of quantification (ULOQ) which was set at 10.0 µg/mL to focus on small concentrations that are expected in the samples. The method validation is shown exemplarily for one of the Arnica STLs, helenalin isobutyrate. First, linearity was evaluated (Figure S2). Calibration curves of the other analytes are shown in Figures S4-S11.

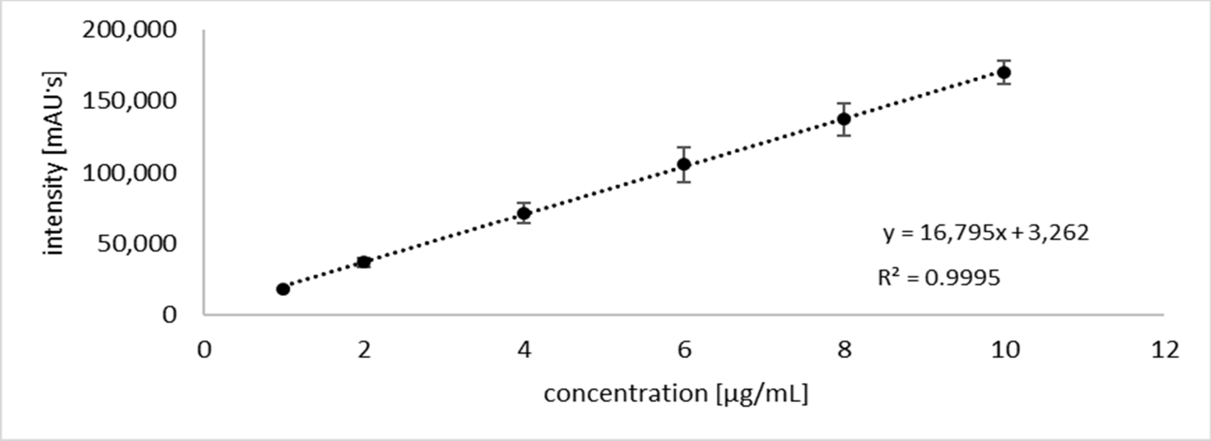

Figure S2.: Calibration curve of helenalin isobutyrate in full method validation.

ICH guideline M10 demands back-calculated concentrations of the calibration standards using slope and intercept of the calibration equation. Results are shown in Table S3. All back-calculated concentrations should be within ± 15% of the nominal concentration (or ± 20% in case of LLOQ) and must be fulfilled for 50% of the calibration standards per concentration level. In this case, three calibration standards were used for each concentration level, thus two of three standards should be within the specifications. In this example, two back-calculated concentrations of replicate 3 (4.66 µg/mL and 7.07 µg/mL) exceed the criterion ± 15% but the other two replicates at the corresponding concentration levels fulfil the specifications. Hence, the criterion is fulfilled.

Table S3. : Back-calculated concentrations of helenalin isobutyrate calibration standards as well as deviations in full validation.

calibration level [µg/mL]	replicate 1 [µg/mL]	deviation	replicate 2 [µg/mL]	deviation	replicate 3 [µg/mL]	deviation
1.00	0.88	12%	0.93	7%	0.90	10%
2.00	2.02	1%	1.77	12%	2.23	11%
4.00	4.00	0%	3.58	11%	4.66	**17%**[1]
6.00	5.90	2%	5.34	11%	7.07	**18%**[1]
8.00	7.71	4%	7.35	8%	8.88	11%
10.00	9.43	6%	9.81	2%	10.57	6%

[1] Single value with deviation >15%, nevertheless, criteria are fulfilled for >50% of the calibration level.

Next, selectivity was evaluated using blank samples. Response of interfering compounds should be ≤ 20% of the analyte response and ≤ 5% of IS response in the LLOQ sample. No significant response is observed at the retention times of the analytes or the IS in the blank samples. Specificity was evaluated by comparing molecular weight and chromatographic separation of structurally similar compounds. Molecular weight of helenalin isobutyrate (332.1617 u) is similar to the molecular weight of 11α,13-dihydrohelenalin isobutyrate (334.1773 u) and helenalin methacrylate (330.1460 u). Helenalin isobutyrate and 11α,13-dihydrohelenalin methacrylate (332.1617 u) are isobaric. For these four analytes and the IS the chromatographic separation was inspected. Retention time differs in 0.399 min (11α,13-dihydrohelenalin isobutyrate), 0.346 min (helenalin methacrylate), 0.745 min (11α,13-dihydrohelenalin methacrylate) and 3.121 min (IS), so that no analytes coelute. Carry-over was evaluated by checking a blank sample measured after the ULOQ. Carry over should be ≤ 20% of the analyte response and ≤ 5% of IS response. Specifications for carry over are fulfilled.

Accuracy and precision should be determined within each run (5 replicates) and between different runs (3 runs) for four concentration levels (LLOQ, within three times of LLOQ (= low QC), 30 -50% of the calibration curve range (= medium QC) and at least 75% of ULOQ (high QC)). At the LLOQ accuracy should be within 80-120% of the nominal concentration. At each other concentration level accuracy should be 85-115% of the nominal concentration. Precision (% coefficient of variation (%CV)) should be at within 80-120% at the LLOQ and 85-115% at other concentration levels. Accuracy and precision are shown in Table S4 (within run) and in Table S5 (between runs). All criteria for accuracy and precision are fulfilled.

Table S4.: Within run accuracy (acc.) and precision (prec.; %CV) of helenalin isobutyrate in full validation determined with five injections (inj.) of each calibration standard. Nominal (nom. c) and calculated (calc. c) concentrations are presented as µg/mL values.

nom. c	calc. c I1	acc. I1	prec. I1	calc. c I2	acc. I2	prec. I2	calc. c I3	acc. I3	prec. I3	calc. c I4	acc. I4	prec. I4	calc. c I5	acc. I5	prec. I5
1.00	1.12	112%	114%	0.92	92%	94%	0.94	94%	96%	0.94	94%	96%	0.97	97%	99%
2.00	1.87	93%	101%	1.78	89%	96%	1.84	92%	100%	1.86	93%	101%	1.88	94%	102%
4.00	3.94	99%	103%	3.66	92%	95%	3.89	97%	101%	3.88	97%	101%	3.82	97%	100%
10.00	9.91	99%	105%	9.16	92%	97%	9.69	77%	103%	9.24	92%	98%	8.98	90%	96%

Table S5.: Between run accuracy (acc.) and precision (prec.; %CV) of helenalin isobutyrate in full validation determined with three injections of the same calibration standard in three independent runs. Nominal (nom. c) and calculated (calc. c) concentrations are presented as µg/mL values.

nom. c	calc. c run 1	acc. run 1	prec. run 1	calc. c run 2	acc. run 2	prec. run 2	calc. c run 3	acc. run 3	prec. run 3
1.00	1.12	112%	110%	0.94	94%	92%	1.00	100%	98%
2.00	1.87	93%	102%	1.83	91%	100%	1.78	89%	98%
4.00	3.94	99%	101%	3.82	96%	98%	3.94	98%	101%
10.00	9.91	99%	104%	9.20	92%	97%	9.41	94%	99%

Matrix effects are evaluated by spiking PBS matrix samples with Arnica tincture. The spiked samples show the typical Arnica sesquiterpene lactone pattern. Blank samples

show no signals in the EICs 245.1172 and 247.1329. Hence, no matrix compound affects the identification of the Arnica sesquiterpene lactones.

1.2 Validation with increased injection volume

Partial method validation was performed because of the modification of our fully validated method. LOD (0.3 ng/mL) and LOQ (1.0 ng/mL) were determined and linearity, selectivity, specificity, accuracy, precision and carry over were evaluated for three analytes. In Figure S3 the linearity of helenalin isobutyrate is shown in a concentration range between 1.0 ng/mL and 100.0 ng/mL.

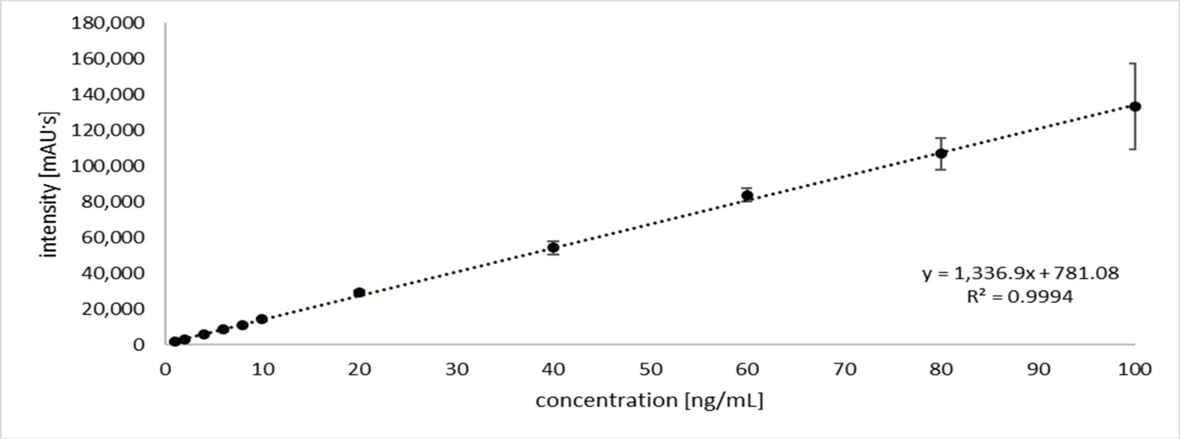

Figure S3.: Calibration curve of helenalin isobutyrate in partial method validation.

Tables S6-S8 give the back-calculated concentrations as well as data on accuracy and precision. Deviations are within ± 15% of the nominal concentration (or ± 20% in case of LLOQ) for at least 50% of the calibration standards per concentration level. Thus, all criteria described in the ICH guideline M10 were fulfilled for the partial validation of the UHPLC-HRMS method.

Table S6.: Back-calculated concentrations of helenalin isobutyrate calibration standards as well as deviations in partial validation.

calibration level [ng/mL]	replicate 1 [ng/mL]	deviation	replicate 2 [ng/mL]	deviation	replicate 3 [ng/mL]	deviation
1.00	0.93	7%	0.93	1%	0.93	7%
2.00	2.15	8%	2.15	7%	2.15	1%
4.00	3.85	4%	3.85	5%	3.85	1%
6.00	6.43	7%	6.43	5%	6.43	3%
8.00	8.20	3%	8.20	3%	8.20	0%
10.00	10.58	6%	10.58	10%	10.58	5%
20.00	19.40	3%	19.40	5%	19.40	1%
40.00	41.60	4%	41.60	8%	41.60	3%
60.00	54.24	10%	54.24	6%	54.24	7%
80.00	80.79	1%	80.79	11%	80.79	11%
100.00	105.25	5%	105.25	9%	105.25	1%

Table S7.: Within run accuracy (acc.) and precision (prec.; %CV) of helenalin isobutyrate in partial validation determined with five injections (I) of each calibration standard. Nominal (nom. c) and calculated (calc. c) concentrations are presented as ng/mL values.

nom. c	calc. c I1	acc. I1	prec. I1	calc. c I2	acc. I2	prec. I2	calc. c I3	acc. I3	prec. I3	calc. c I4	acc. I4	prec. I4	calc. c I5	acc. I5	prec. I5
1.00	1.09	109%	97%	1.18	118%	104%	1.19	119%	105%	1.13	113%	100%	1.07	107%	94%
2.00	1.99	99%	97%	1.96	98%	96%	2.05	103%	100%	2.14	107%	105%	2.10	105%	102%
40.00	41.38	103%	98%	42.56	106%	101%	42.92	107%	101%	39.35	98%	93%	45.27	113%	107%
80.00	88.82	111%	105%	83.12	104%	98%	83.73	105%	99%	87.93	110%	104%	80.39	100%	95%

Table S8.: Between run accuracy (acc.) and precision (prec.; %CV) of helenalin isobutyrate in partial validation determined with three injections of the same calibration standard in three independent runs. Nominal (nom. c) and calculated (calc. c) concentrations are presented as ng/mL values.

nom. c	calc. c run 1	acc. run 1	prec. run 1	calc. c run 2	acc. run 2	prec. run 2	calc. c run 3	acc. run 3	prec. run 3
1.00	0.93	93%	93%	1.01	101%	101%	1.07	107%	101%
2.00	2.40	**120%**[1]	115%	1.86	93%	89%	1.99	99%	89%
40.00	37.67	94%	104%	33.49	84%	92%	37.47	94%	92%
80.00	64.60	81%	101%	56.72	71%	88%	71.02	89%	88%

[1] Single value with deviation >115%, nevertheless, criteria are fulfilled for >50% of the calibration level.

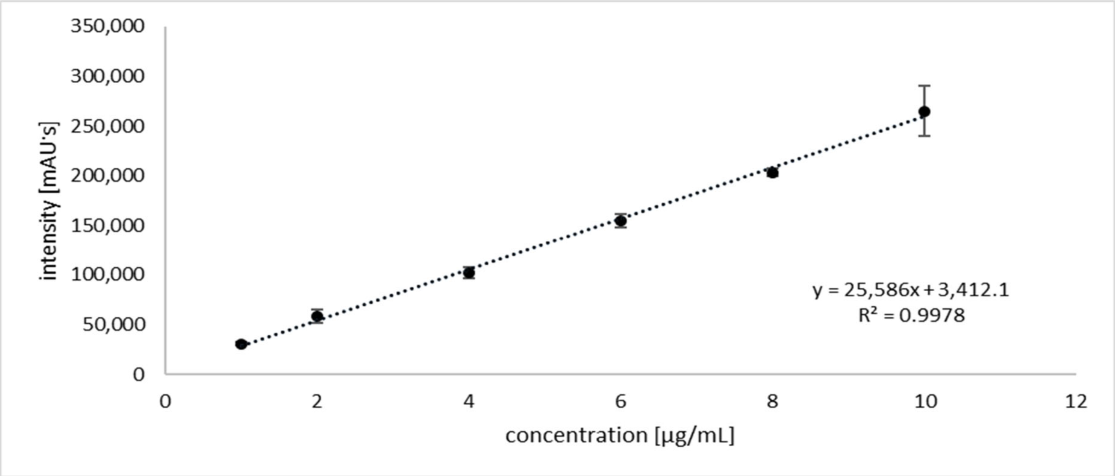

Figure S4.: Calibration curve of DHac in full method validation.

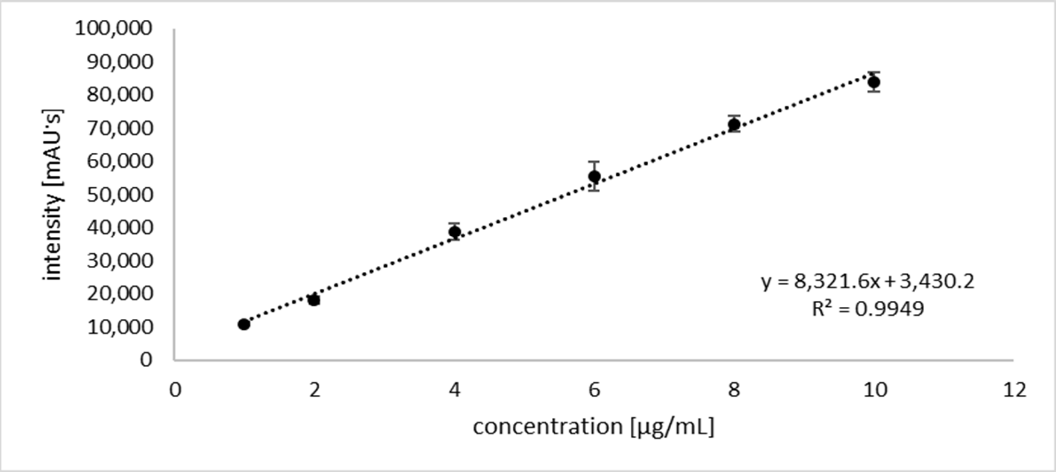

Figure S5.: Calibration curve of DHib in full method validation.

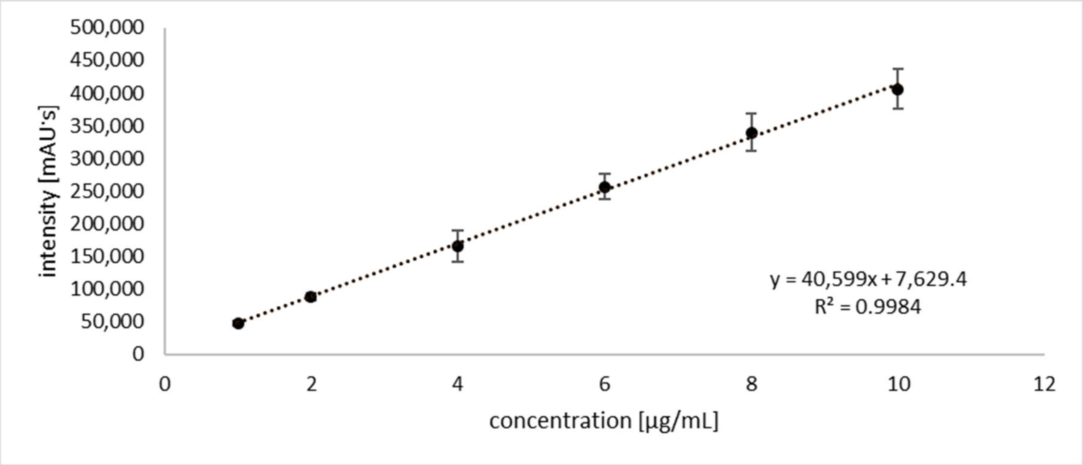

Figure S6.: Calibration curve of DHmb in full method validation.

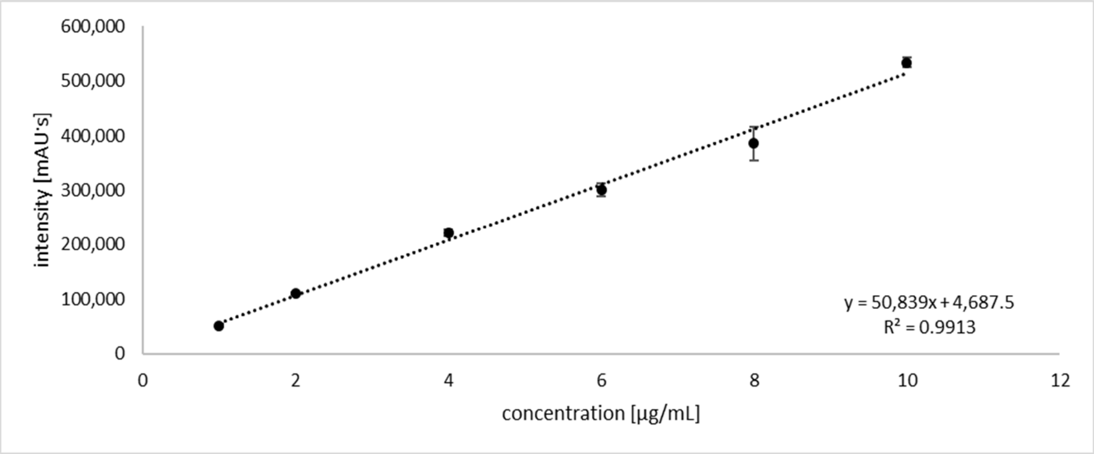

Figure S7.: Calibration curve of DHt in full method validation.

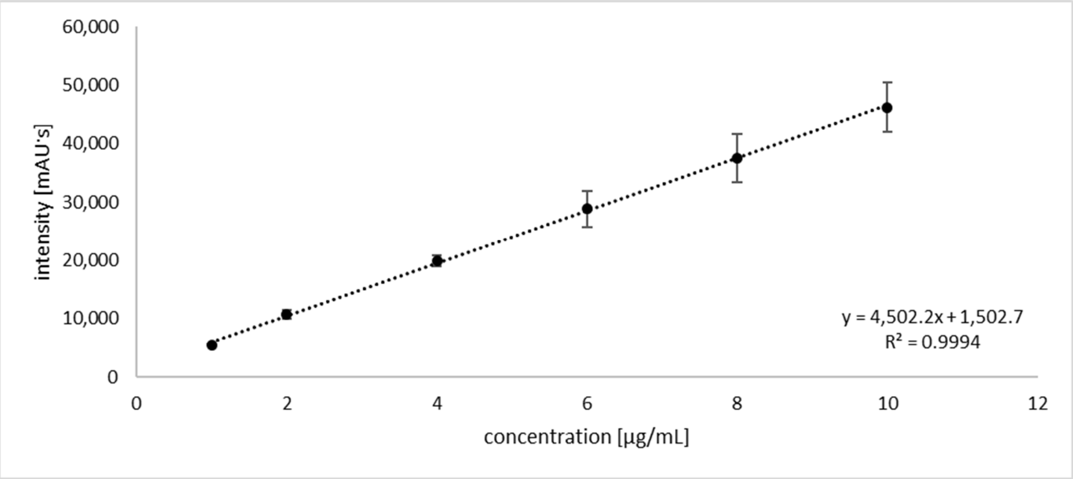

Figure S8.: Calibration curve of H in full method validation.

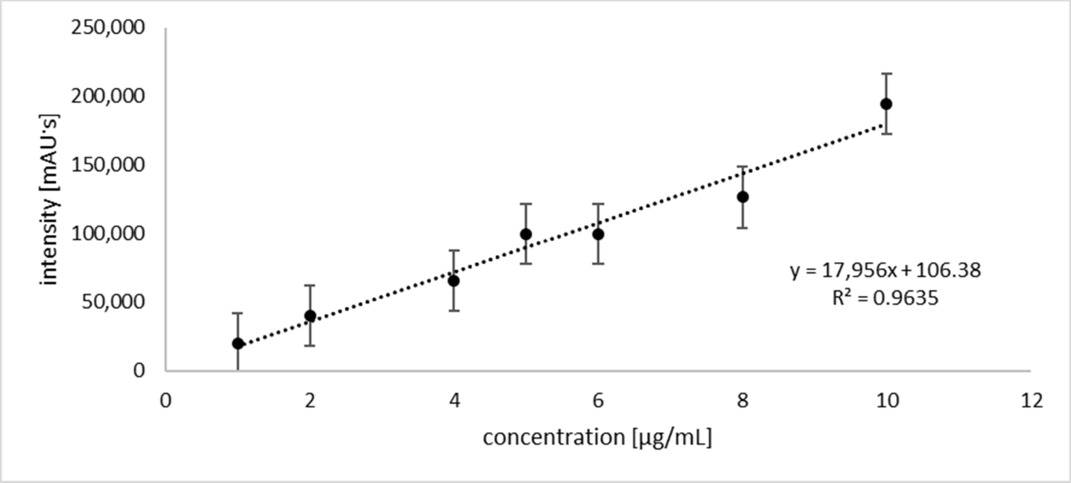

Figure S9.: Calibration curve of Hac in full method validation.

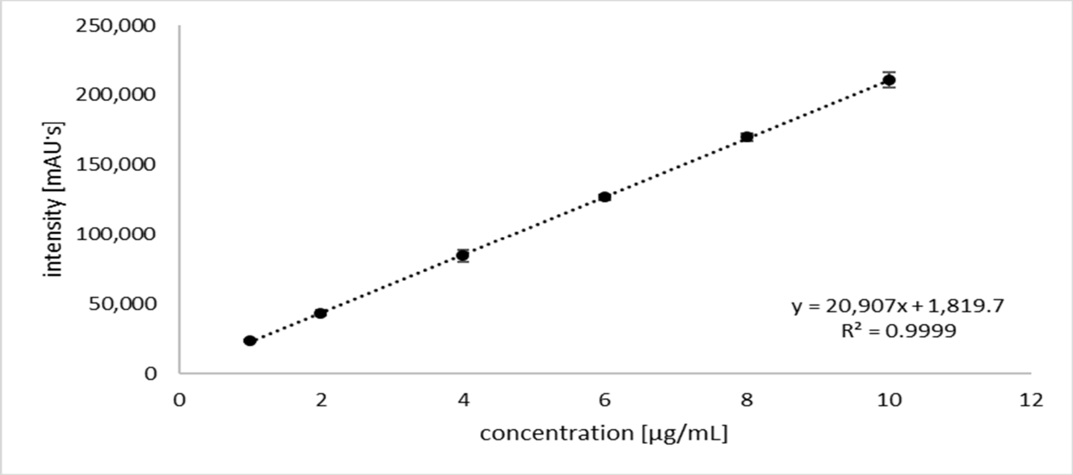

Figure S10.: Calibration curve of Hma in full method validation.

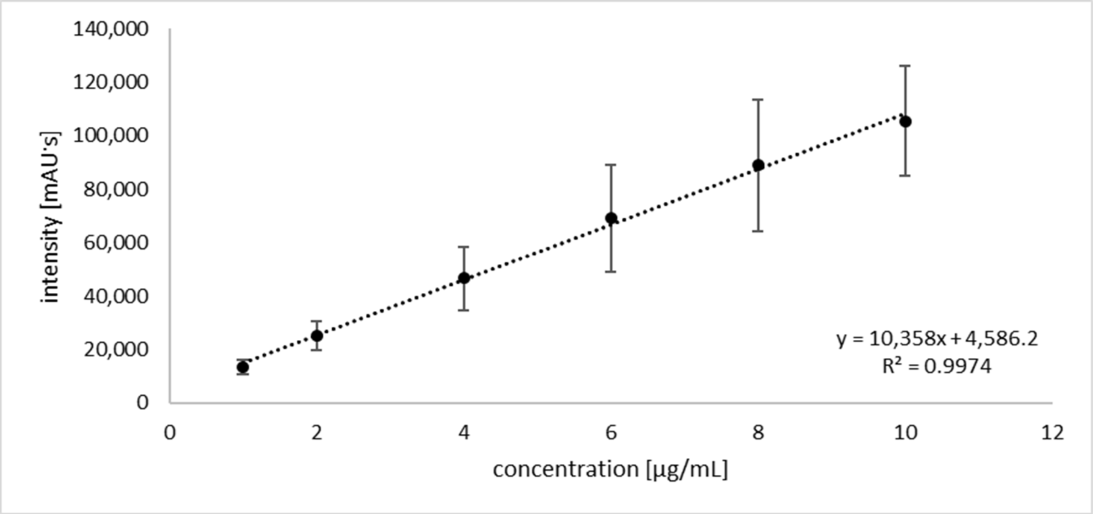

Figure S11.: Calibration curve of Ht in full method validation.

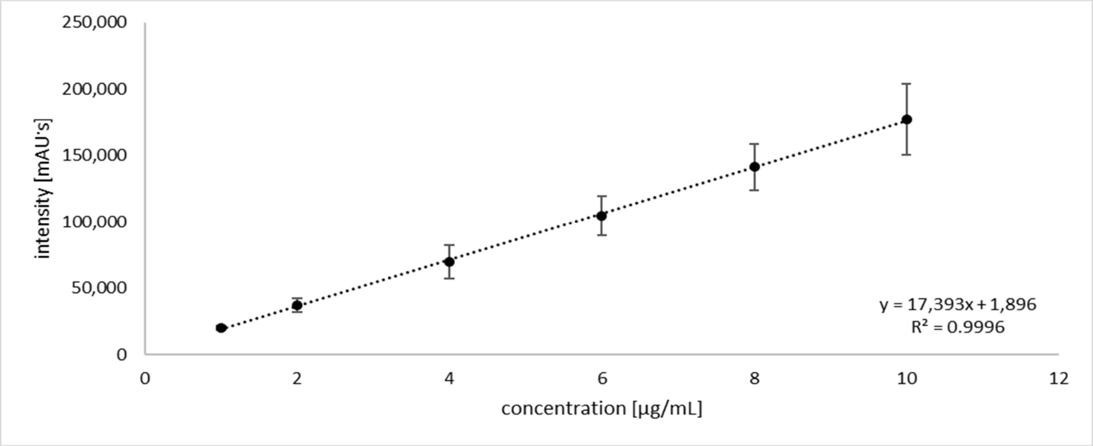

Figure S12.: Calibration curve of Hmb in full method validation.

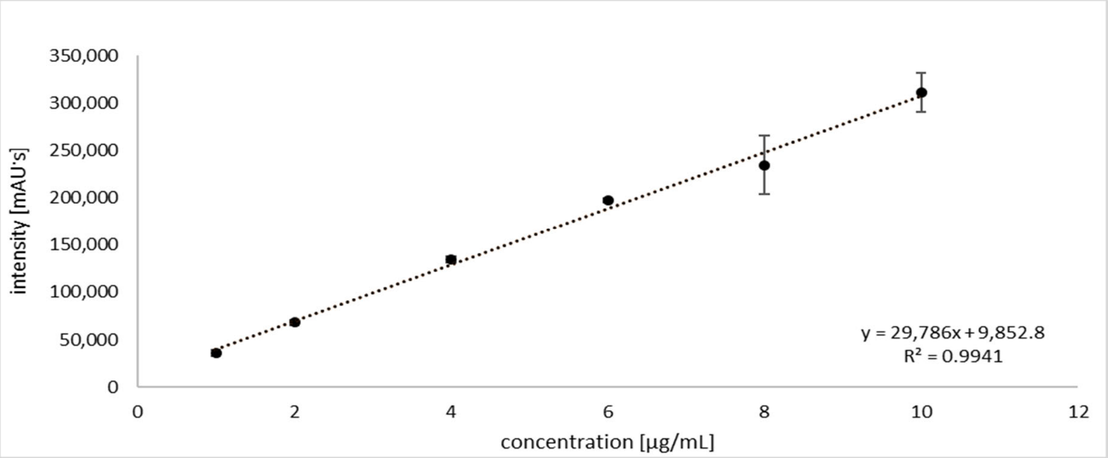

Figure S13.: Calibration curve of DHma in full method validation.
